# Adherence to the Eat-Lancet diet and its association with depression and anxiety among Iranian adults: a cross-sectional multicentric study

**DOI:** 10.3389/fnut.2025.1524652

**Published:** 2025-03-27

**Authors:** Ghazaal Alavi Tabatabaei, Noushin Mohammadifard, Fahimeh Haghighatdoost, Hamed Rafiee, Mehdi Abbasi, Farid Najafi, Hossein Farshidi, Masoud Lotfizadeh, Tooba Kazemi, Hamidreza Roohafza, Nizal Sarrafzadegan

**Affiliations:** ^1^Isfahan Cardiovascular Research Center, Cardiovascular Research Institute, Isfahan University of Medical Sciences, Isfahan, Iran; ^2^Interventional Cardiology Research Center, Cardiovascular Research Institute, Isfahan University of Medical Sciences, Isfahan, Iran; ^3^Hypertension Research Center, Isfahan Cardiovascular Research Institute, Isfahan University of Medical Sciences, Isfahan, Iran; ^4^Research Center for Environmental Determinants of Health, Health Institute, Kermanshah University of Medical Sciences, Kermanshah, Iran; ^5^Rajaie Cardiovascular Medical and Research Center, Iran University of Medical Sciences, Tehran, Iran; ^6^School of Health, Shahrekord University of Medical Sciences, Shahrekord, Iran; ^7^Cardiovascular Diseases Research Center, Birjand University of Medical Sciences, Birjand, Iran; ^8^Cardiac Rehabilitation Research Center, Cardiovascular Research Institute, Isfahan University of Medical Sciences, Isfahan, Iran

**Keywords:** Eat-Lancet diet, mental health, depression, anxiety, mental disorders

## Abstract

**Background:**

Previous studies have shown a connection between diet and mental health. However, there is limited evidence on how emerging diets, particularly the EAT-Lancet reference diet (ELD), relate to depression and anxiety. This study aims to investigate the potential impact of ELD adherence on these mental health conditions.

**Methods and materials:**

This cross-sectional study recruited 1,970 Iranian adults using a stratified multistage random cluster sampling method, part of a community-based investigation conducted in five cities from February 2018 to July 2019. Participants’ regular dietary intake was assessed using a validated food frequency questionnaire. The ELD was formulated based on the consumption of the 14 dietary components outlined in the ELD. Anxiety and depression were evaluated using a validated Iranian version of the Hospital Anxiety and depression Scale.

**Results:**

In the fully adjusted model, there was no significant association between ELD adherence and depressive symptoms (aOR_T3 vs. T1_ = 0.89; 95% CI: 0.66, 1.19; *p* trend = 0.42) and anxiety (aOR _T3 vs. T1_ = 0.93; 95% CI: 0.70, 1.23; *p* trend = 0.62) in the whole population. In stratified analysis by sex, a significant inverse association was found only between ELD and depression among men (aOR _T3 vs. T1_ = 0.66; 95% CI: 0.40, 1.07; *p* trend = 0.047) but not women (aOR = 0.71, 95% CI: 0.44, 1.15; *p* trend = 0.103). No significant association was observed between ELD and anxiety either in men or women.

**Conclusion:**

Despite a null association between ELD and depressive symptoms and anxiety in the whole population, higher adherence to ELD was associated with a lower risk of depressive symptoms in males.

## Introduction

Nearly 450 million individuals worldwide grapple with mental health conditions, particularly depression and anxiety (1). These disorders, owing to their chronic nature, impose a substantial burden to healthcare system and contribute significantly to disability, emerging as one of the primary causes of disability-adjusted life years (DALYs) on a global scale (2). Depression and anxiety are intricate conditions influenced by a combination of genetic predispositions and lifestyle factors (3). Psychotherapies and antidepressant medications, such as selective serotonin reuptake inhibitors, are the key treatment in major depressive disorders. Nonetheless, approximately 30% of individuals with depression do not respond adequately to antidepressants. There has been an increased interest in pivotal impact of lifestyle especially eating habits (4, 5).

Within the spectrum of healthy lifestyle, the advantageous effects of a healthy diet have garnered considerable attention (5–7). However, the interpretation of what constitutes a healthy diet—whether it excludes all animal products or primarily emphasizes plant-based foods—often leads to conflicting conclusions (5, 7–9). In this context, the international EAT-Lancet commission, devised a global planetary healthy reference diet in 2019 (10, 11). It is structured based on daily energy intake and designed to be adaptable to various preferences, environments, and populations. This diet is low in saturated fat and high in fiber, plant-based food and restricts highly processed food and animal products (12). Previous research has predominantly concentrated on the effects of this eating plan on chronic conditions, notably cardiovascular disease and cancer (9, 13, 14). The outcomes yielded conflicting perspectives regarding the efficacy of the Eat-Lancet diet (ELD) (9, 15–17). For instance, the EPIC-Oxford study suggested a favorable impact of ELD on ischemic heart disease and diabetes (15). In contrast, findings from the NutriNet-Santé cohort indicated a correlation between higher adherence to the ELD and reduced cancer risk solely among females, but no significant association between the ELD and cardiovascular disease (9). However, evidence remains scarce concerning the ELD-mental health disorders relationship. In a study involving 180,446 participants from the UK Biobank, a pronounced adherence to the ELD was correlated with a diminished risk of experiencing depression, anxiety, and co-occurrence (18). Similar results emerged from a recent cross-sectional study carried out in Mashhad, Iran, regarding depression; however, no associations were found between the diet and anxiety or stress (19).

Moreover, it is noteworthy that the EAT-Lancet planetary health diet might lack in several essential micronutrients (12). Relying primarily on healthy plant-based foods while minimizing animal-derived sources might be associated with increased risk of mental disorders (20, 21) since it does not guarantee sufficient nutrient provision, especially crucial minerals like iron, calcium, and zinc, which play pivotal roles in the onset and progression of psychological disorders (12). In addition, mounting evidence has shown substantial differences in prevalence, symptoms, age of onset, and prognosis of mental disorders between men and women (22–24). Consequently, there is an imperative need to assess the health impacts of this emerging diet, particularly in relation to mental health disorders such as depression and anxiety and examine sex-specific associations. Hence, the current study aimed to explore the correlation between the ELD and the risk of depression and anxiety within a multi-center sample of Iranian females and males to shed light on this crucial aspect of dietary impact.

## Methods

### Study population

This cross-sectional investigation was performed using the baseline data of the Knowledge And Practice Of dyslipidemia prevention, management, and control (LIPOKAP) national study, which is a community-based trial. The LIPOKAP study was conducted between February 2018 and July 2019 in five cities in Iran: Isfahan, Birjand, Bandar Abbas, Kermanshah, and Shahrekord (25). However, to increase the power of study, a total of 2,456 adults aged 18 and above were enrolled through a stratified multistage random cluster sampling method. Participants were recruited from health care centers and selected randomly based on population distribution. The sample size calculation utilized a stratified multistage random cluster sampling approach. Initially, an appropriate sample size was calculated, then multiplied owing to the presence of diverse clusters. Based on the suggested formula for cross-sectional studies with prevalence ([(Z 1-*α*/2)^2^ * P * (1-P)] / d^2^), we calculated adequate sample size. Considering a prevalence of 15% for depression in Iranians (26, 27), type I error (α) = 5% and d = 0.03 (one-fifth of the prevalence), a sample size of 544 was estimated. The final sample size for each city was established by considering population distribution within cities and their urban–rural divides. Available clusters within healthcare centers were randomly chosen, and specific sample sizes were allocated to each cluster based on the population distribution. Subsequently, participants were randomly selected according to this allocation. Trained interviewers invited eligible participants after random selection. Subjects were included if they were seemingly healthy with no chronic disease and not been on any medications regimen. Exclusion criteria encompassed various systemic or dyslipidemia-related diseases, chronic kidney or liver disease, cancer, immune system disorders, and under- or over-estimation of energy intake (< 800 or > 4200 Kcal/day). We further excluded individuals who were adhering to any special diets for weight loss or chronic disease management. The final analysis included 1994 eligible participants after applying exclusion criteria. Ethics approval was obtained from the ethics committee of the Isfahan University of Medical Sciences (protocol number: IR.MUI.RC. 1395.4.077), and all participants provided a written informed consent.

### Data collection

Sociodemographic information and physical activity levels were collected through predefined questionnaires and the International Physical Activity Questionnaire (IPAQ), respectively. Physical activity level was calculated as the metabolic equivalent (MET) hour/week (28, 29). An interviewer-administered questionnaire was utilized to evaluate socio-demographic and lifestyle characteristics such as age, gender, smoking habits, and socioeconomic status (SES) (30). Smoking, alcohol consumption and addiction were assessed by Global adult Tobacco Survey (GATS (31)) questionnaire and validated questionnaire, respectively (32). More details about the study design, participants, and socioeconomic assessment can be found in the methodological study description (25).

### Usual dietary intake

A validated 110-item semi-quantitative food frequency questionnaire (FFQ) was used to evaluate the usual dietary habits of participants over the past year (33). This questionnaire assessed each food item based on typical portion sizes, offering nine frequency categories, ranging from never/seldom to >6 times/day. Using the portion size and consumption frequency data, the average intake of each food item (in grams per day) was estimated for all participants. Subsequently, energy and nutrient intakes were computed using Nutritionist IV software adjusted for Iranian foods.

### Computation of eat lancet diet (ELD)

ELD was assessed based on the following equation and detailed explanations have been described elsewhere (11):


$$\begin{array}{l} ELD-Ij=\\ \frac{100\times \left\{\sum_{\mathrm{componen}t_{i=1}}^{14}\frac{a_{i}\times \left(\mathrm{cut}-\mathrm{of}f_{i}-\frac{\mathrm{consumptio}n_{ij}\times 2500}{\mathrm{Energy}\;\mathrm{intak}e_{j}}\right)}{\mathrm{cut}-\mathrm{of}f_{i}}\right\}}{14} \end{array}$$

In this context, “i” denotes the EAT-Lancet food groups, while “j” represents individuals. The value “ai” is assigned as 1 to denote components meant for restriction and −1 to indicate components for promotion. The computation of the ELD generates a continuous variable, which can be either positive or negative. A higher score signifies a stronger adherence to the ELD reference. ELD components and cut-offs for scoring method are shown in detail in Supplementary Table 1.

### Mental health assessment

A verified Iranian adaptation of the Hospital Anxiety and Depression Scale (HADS) was used to evaluate anxiety and depression levels (34). This questionnaire consists of two sections, each containing seven items rated on a four-point scale. Possible scores ranged from 0 to 21, with lower scores indicating less anxiety or depressive symptoms and higher scores indicating greater severity. Scores of 7 or below were deemed normal (indicating minimal to no symptoms of depression or anxiety), while scores of 8 or higher suggested the presence of depression or anxiety.

### Statistical analysis

Participants were categorized into three groups based on the tertiles of ELD. Mean and standard deviations, along with percentages were employed to summarize continuous and categorical variables, respectively. One-way analysis of variance (ANOVA) was utilized to compare means across different tertiles of ELD while percentages were compared using Chi-square test. To account for the center effect, we incorporated a random effects model into our analysis. This adjustment allows us to consider the variability between different centers and its impact on the outcomes measured. Multiple logistic regression was utilized to estimate adjusted odds ratios (aORs) and 95% confidence intervals (CIs) for depression and anxiety across the tertiles of ELD in crude and multivariable adjusted models. Cofounders were selected based on determinant factors of mental health status based on literature or different distribution of demographic and lifestyle variables across tertiles of ELD in our study population. The first adjusted model was controlled for the confounding effects of age, sex, and energy, while the second model was additionally adjusted for education, socioeconomic status, marital status, smoking, and physical activity. The linear trend across tertiles of ELD was estimated as a continuous variable in the logistic regression model. Crude and multivariable aORs and 95% CIs for the severity of depression and anxiety were calculated by ordinal logistic regression all statistical analyses were performed using the Statistical Package for Social Sciences (SPSS Inc., version 20), with significance set at *p* < 0.05 for all analyses.

## Results

### Demographic characteristics

The study population was comprised of 1994 individuals with a mean (SD) age of 39.79 ± 13.87 years. Over 50 % of participants were female and the ELD scores ranged from −160.44 to 105.63. Table 1 demonstrates the general characteristics of study population over tertiles of ELD score. Participants in the highest tertile of ELD score were more likely to be female and physically active, and had a higher marriage rate than those in the lowest tertile. They were also older and less likely to be current smokers.

**Table 1:** General characteristics of participants based Eat-Lancet diet groups^1^.

	Eat-Lancet diet (*N* = 1994)			*p* value^2^
	Tertile 1 (−160.44, −9.81)	Tertile 2 (−9.77, 10.06)	Tertile 3 (10.15, 105.63)	
*n*	664	665	665	
Age (years)	37.60 ± 13.60	40.41 ± 14.05	41.38 ± 13.70	<0.001
Physical activity (MET.h/wk)	3176.8 ± 4456.8	2,831 ± 3524.3	3340.5 ± 3936.3	0.006
Female (%)	45.6	50.8	60.4	<0.001
Married (%)	79.4	82.9	85.3	0.006
Socioeconomic status (%)				0.459
Low	9.3	9.3	11.3	
Middle	40.4	43.8	41.7	
High	50.3	46.9	47.1	
Education year (%)				0.271
0–5 y	23.9	23.3	19.3	
6–12 y	46.4	46.5	45.8	
≥13 y	29.7	30.3	34.9	
Current smoker (%)	15.1	11.4	7.8	<0.001
Depression	4.40 ± 3.66	4.51 ± 3.55	4.37 ± 3.48	0.74
Anxiety	4.65 ± 3.75	4.70 ± 3.81	4.67 ± 3.81	0.97
Eat-Lancet score	−29.28 ± 17.43	0.21 ± 5.52	26.67 ± 14.86	<0.001

^1Data reported are mean ± SD otherwise indicated.^

^2Chi-square test was used for categorical variables and one-way ANOVA for continuous variables.^

### Dietary intake

The dietary intakes of participants across the tertiles of ELD score are shown in Table 2. Compared with the first tertile, those in the third tertile consumed greater amounts of whole grains, vegetables, legumes, fruits, nuts, protein and fiber. Conversely, they consumed lower amounts of potatoes, red meat, eggs, added sugar, saturated fats, monounsaturated fat (MUFA) and added sugar (p for trend <0.001 for all these groups). We did not observe any significant relationship between the tertiles of ELD score and seafood, poultry and dairy intake.

**Table 2:** Dietary intakes of study participants based on Eat-Lancet diet groups.^1^

	Eat-Lancet diet (*N* = 1994)			*p* trend^2^
	Tertile 1 (*n* = 664)	Tertile 2 (*n* = 665)	Tertile 3 (*n* = 665)	
Whole grain (g/day)	73.16 ± 3.71	102.02 ± 3.69	113.76 ± 3.71	< 0.001
Potato (g/day)	42.0 ± 1.19	34.94 ± 1.18	27.65 ± 1.19	< 0.001
Vegetables (g/day)	265.30 ± 4.44	283.36 ± 4.41	358.91 ± 4.43	< 0.001
Red meat (g/day)	38.31 ± 0.93	31.93 ± 0.92	27.10 ± 0.92	< 0.001
Sea foods (g/day)	14.76 ± 0.61	14.79 ± 0.60	16.42 ± 0.61	0.091
Egg (g/day)	30.26 ± 0.81	20.63 ± 0.80	17.40 ± 0.81	< 0.001
Legumes (g/day)	28.51 ± 1.17	33.94 ± 1.17	41.54 ± 1.17	< 0.001
Fruits (g/day)	178.31 ± 4.49	226.31 ± 4.47	346.59 ± 4.49	< 0.001
Nuts (g/day)	16.89 ± 0.87	22.14 ± 0.86	34.90 ± 0.87	< 0.001
Poultry (g/day)	41.60 ± 1.23	42.23 ± 1.22	40.46 ± 1.23	0.587
Added sugar (g/day)	103.78 ± 1.80	48.20 ± 1.79	30.90 ± 1.80	< 0.001
Unsaturated fat (g/day)	23.34 ± 0.68	23.52 ± 0.68	22.58 ± 0.68	0.585
Saturated fat (g/day)	4.65 ± 0.23	3.05 ± 0.23	2.48 ± 0.23	< 0.001
Dairy (g/day)	235.81 ± 6.71	244.95 ± 6.68	241.25 ± 6.71	0.628
Eat-Lancet score	−29.28 ± 17.43	0.21 ± 5.52	26.67 ± 14.86	<0.001
Carbohydrate	277.93 ± 1.66	284.17 ± 1.65	276.64 ± 1.65	003
Protein	90.17 ± 0.65	94.65 ± 0.65	95.34 ± 0.65	< 0.001
Fiber	19.63 ± 0.17	22.13 ± 0.17	25.42 ± 0.17	< 0.001
PUFA	25.99 ± 0.36	24.95 ± 0.36	26.8 ± 0.36	0.001
SFA	34.34 ± 0.45	31.4 ± 0.44	32.56 ± 0.44	0.001
MUFA	29.02 ± 0.3	26.54 ± 0.3	27.31 ± 0.3	< 0.001
Fat	93.52 ± 0.81	88.25 ± 0.8	89.95 ± 0.8	< 0.001
Added sugar	103.9 ± 1.8	48.68 ± 1.78	30.72 ± 1.78	< 0.001

^1Values are mean ± SE and adjusted for age, sex, and energy.^

^2Derived from ANCOVA.^

### Association with mental health outcomes

Table 3 presents mean and standard errors (SE) of depression and anxiety scores in crude and adjusted models across the tertiles of ELD score. No significant linear trend was observed for the scores of depression and anxiety over the tertiles of ELD score.

**Table 3:** Mean of depression and anxiety scores based on Eat-Lancet diet groups.^1^

	Eat-Lancet diet (*N* = 1994)			*p* trend^2^
	Tertile 1 (*n* = 664)	Tertile 2 (*n* = 665)	Tertile 3 (*n* = 665)	
Depression				
Crude	4.39 ± 0.14	4.51 ± 0.13	4.36 ± 0.13	0.740
Model 1	4.55 ± 0.14	4.54 ± 0.14	4.18 ± 0.14	0.098
Model 2	4.52 ± 0.14	4.56 ± 0.13	4.19 ± 0.14	0.118
Anxiety				
Crude	4.65 ± 0.14	4.7 ± 0.14	4.67 ± 0.14	0.970
Model 1	4.85 ± 0.15	4.66 ± 0.14	4.50 ± 0.15	0.234
Model 2	4.86 ± 0.15	4.69 ± 0.14	4.48 ± 0.15	0.194

^1Values are mean ± SE.^

^2^Derived from Analysis of Covariance (ANCOVA).

Model 1: Adjusted for age, sex, and energy.

Model 2: Adjusted as for model 1 plus marital status (category), smoking (category), physical activity, socioeconomic status and center effect.

Table 4 provides the crude and multivariable-adjusted aORs (95% CIs) for the odds of depression and anxiety across tertiles of ELD score. After adjusting for age, sex, and energy (model1), the odds of depression was significantly lower in the third tertile of ELD score compared with the first tertile (aOR = 0.75, 95% CI: 0.56, 1.0; *p* trend = 0.098). However, this association was not significant after adjusting for additional variables in model 2 (aOR = 0.78, 95% CI: 0.57, 1.05; *p* trend = 0.118). No significant associations were observed between ELD and the odds of anxiety in the crude or adjusted models.

**Table 4:** Crude and multivariable-adjusted odds ratios and 95% CIs for anxiety and depression based on Eat-Lancet diet groups.

	Eat-Lancet diet (*N* = 1994)			*p* trend^1^
	Tertile 1 (*n* = 664)	Tertile 2 (*n* = 665)	Tertile 3 (*n* = 665)	
Depression				
Crude	1	0.98 (0.75, 1.29)	0.93 (0.71, 1.22)	0.614
Model 1	1	0.91 (0.69, 1.21)	0.75 (0.56, 1.00)	0.051
Model 2	1	0.95 (0.71, 1.27)	0.78 (0.57, 1.05)	0.096
Anxiety				
Crude	1	1.07 (0.83, 1.40)	1.04 (0.80, 1.36)	0.748
Model 1	1	0.94 (0.71, 1.23)	0.83 (0.63, 1.10)	0.198
Model 2	1	0.94 (0.71, 1.25)	0.81 (0.61, 1.09)	0.159

^1Derived from Mantel–Haenszel test.^

Model 1: Adjusted for age, sex, and energy.

Model 2: Adjusted as for model 1 plus marital status (category), smoking (category), physical activity, socioeconomic status and center effect.

Tertile 1 is the reference group for all odds ratios.

### Gender-based stratification

Table 5 shows the crude and adjusted aORs for depression and anxiety over tertiles of ELD score, stratified by sex. Among men, the odds of depression was significantly lower in the third tertile compared with the first tertile in both crude (aOR = 0.62, 95% CI: 0.39, 1.00; *p* trend = 0.019) and fully-adjusted model (aOR = 0.64, 95% CI: 0.38, 1.06; *p* trend = 0.049). No significant association was observed for anxiety in any of the models. Among women, we found a null association between ELD and depression and anxiety in all three models.

**Table 5:** ORs and 95% CI of depression and anxiety across tertiles of Eat-Lancet diet stratified by sex.

	Eat-lancet diet (*N* = 1994)			*p* trend^1^
	Tertile 1	Tertile 2	Tertile 3	
Men				
Depression				
Crude	1	0.45 (0.28, 0.73)	0.62 (0.39, 1.00)	0.019
Model 1	1	0.46 (0.28, 0.74)	0.57 (0.35, 0.92)	0.009
Model 2	1	0.51 (0.31, 0.85)	0.64 (0.38, 1.06)	0.049
Anxiety				
Crude	1	0.54 (0.34, 0.86)	0.74 (0.47, 1.17)	0.116
Model 1	1	0.49 (0.30, 0.79)	0.68 (0.43, 1.09)	0.063
Model 2	1	1.67 (1.01, 2.75)	0.76 (0.44, 1.31)	0.290
Women				
Depression				
Crude	1	1.46 (1.02, 2.10)	1.05 (0.73, 1.50)	0.954
Model 1	1	1.38 (0.95, 1.99)	0.94 (0.65, 1.37)	0.592
Model 2	1	1.35 (0.95, 2.02)	0.92 (0.63, 1.35)	0.510
Anxiety				
Crude	1	1.47 (1.04, 2.08)	1.08 (0.77, 1.51)	0.830
Model 1	1	1.37 (0.96, 1.93)	0.99 (0.70, 1.40)	0.814
Model 2	1	1.03 (0.72, 1.47)	1.41 (1.02, 1.96)	0.772

^1Derived from Mantel–Haenszel test.^

Model 1: Adjusted for age and energy.

Model 2: Adjusted as for model 1 plus marital status (category), smoking (category), physical activity, socioeconomic status and center effect.

Tertile 1 is the reference group for all odds ratios.

### Association with severity of depression and anxiety

Multivariable- aORs for the severity of depression and anxiety are shown in Supplementary Table 2. After adjusting for age, gender, and energy in Model 1, a higher ELD score was linked to a lower risk of experiencing severe depressive symptoms (aOR = 0.74, 95% CI: 0.56, 0.99; *p* trend = 0.040). When controlling for all potential confounders, this trend remained evident but with diminished statistical significance (aOR = 0.76, 95% CI: 0.56, 1.02; *p* trend = 0.071). However severity of anxiety was not related to the ELD score.

## Discussion

Our study revealed no significant association between ELD adherence and depression in the whole population. However, in stratified analysis by sex, we observed that males with higher scores of ELD had 34% lower risk of depression than those with the lowest adherence, while no association was observed in females. Importantly, we noted non-linear trends in depression scores across different tertiles, suggesting that dietary adherence may not yield uniform benefits across all levels of adherence. Moreover, a higher adherence to ELD was associated with a reduction in the severity of depressive symptoms. ELD was not pertinent to the risk of anxiety either in the whole population or in males and females separately.

To the best of our knowledge, this investigation represents one of the few attempts to assess the association of the ELD with the risk of mental health conditions. Prior research has established the protective effects of ELD on the progression of cardiovascular diseases (14, 16), diabetes (17, 35), cancer, and all-cause mortality (9, 13). Nevertheless, our study failed to reveal any significant association between ELD and depression in the whole population; however, it indicated that adherence to ELD among male participants exhibits an inverse non-linear trend with respect to depression.

The ELD diet serves as a key framework for integrating sustainability into national dietary guidelines of diverse cultures, aiming to transform global food production and waste management while improving human health (10). In contrast with earlier studies suggesting beneficial impacts for ELD, there is some evidence suggesting that vegetarian and plant-based diets might be insufficient to meet daily nutrients requirement and thereby increase the risk of mood disorders (20) or at least have no substantial impact (5). In our earlier analysis of this population, we failed to find any relationship between plant-based diet and healthy plant-based diet and mental disorders (5). Consistently, Young showed that individuals with higher adherence to the ELD had lower intake of protein, selenium, zinc, iron, and folate and were more probably to have a poor mood (36). In addition, it is suggested that determining a minimum intake of various food groups for ELD would be able to provide adequate intake of several nutrients (37) and therefore increase diet quality. Indeed, the scoring system used in the ELD study established minimum intake levels for various dietary components at zero grams per day, potentially resulting in deficiencies for micronutrient crucial for optimal brain function, which could subsequently exacerbate depressive symptoms (10). Therefore, it is possible that the non-significant association in our study population and smaller risk reduction in tertile 3 in comparison with tertile 2 in males might be attributed to lower nutrient-density of higher scores of the ELD.

Consistent with our sex-specific association, a French cohort study reported that higher adherent to the Mediterranean diet could significantly reduce depression in males, but not females (38). Apart from variations in risk factors for depressive symptoms between genders and higher reports of depressive symptoms among females (39), this distinction could arise from varying responses to dietary adherence, influenced by different gene expressions among males and females (38, 40, 41). This could potentially result in less benefits from plant-based diet interventions in the mental health of women. Lastly, Hanley Cook et al. previously discussed the issue of micronutrient insufficiency in women following ELD (37). Disparities noted among men and women based on factors such as height, weight, physical activity, as well as the impacts of pregnancy and breastfeeding can lead to a heightened prominence of micronutrient insufficiency among women, suggesting that adhering to ELD may not yield significant benefits in comparison to men.

The null association found in the whole population might be due to the residual or unmeasured variables which can potentially affect mental health status. Moreover, the low or moderate scores of depression in our study population may be another explanation for the null association in the whole population. On the other hand, it is possible that ELD can affect depression risk in populations with higher severity of depression. However, the favorable association found in males might be explained by some reasons. Earlier studies have demonstrated that mental health disorders are complex and multifaceted conditions that could stem from diverse potential mechanisms, including oxidative stress, inflammation, and disrupted neurotransmitter synthesis (3, 42). Moreover, the interconnectedness observed between these disorders and other conditions, such as obesity and insulin resistance, might be due to chronic low-grade inflammation (43, 44). Unhealthy dietary patterns characterized by abundant refined grains, sugary desserts and beverages, along with inadequate intake of fruits and vegetables might trigger elevated levels of pro-inflammatory cytokines (3, 5, 45, 46). Subsequently, this elevation could disrupt the immune system, impact the functioning of neurotransmitters like serotonin, dopamine, glutamate signaling, and the brain-derived neurotrophic factor, altogether play significant roles in psychological disorders specifically depression (3, 5, 47). An alternative explanation could be that ELD and other similar plant-based diets that are enriched in fiber may induce more favorable gut microbiota which can directly attenuate depressive symptoms (6, 48).

There exists a divergence in findings regarding the impact of a nutritious diet on anxiety (5, 7, 48–50). Our study demonstrated a null association between different levels of ELD adherence and the risk of anxiety. Similarly, a recent systematic review and meta-analysis of observational studies failed to show any noteworthy alleviate in anxiety or stress symptoms among individuals adhering to a vegetarian diet (7). Contrary to these results, a body of research suggests that Mediterranean and plant-based diets have beneficial effect on anxiety, particularly in individuals with moderate to severe symptoms (48, 49). Furthermore, a recent study involving participants from the UK Biobank found that adherence to ELD was associated with a reduced occurrence of depression, anxiety, and their co-occurrence (18). These mixed findings, could be potentially arisen from the disparities in the amount and quality of healthy diets among different nations and their genetic vulnerability to mood disorders (4, 51). For instance, Iranians’ diet mainly consists of white rice and bread, along with certain types of fats like n-6 polyunsaturated fatty acids (52). Consuming high levels of refined carbohydrate and these fats promotes the generation of free radicals, leading to increased oxidative stress and inflammation. These factors disrupt brain function and may contribute to the development of anxiety (4).

The results reported in this study should be interpreted in light of the following limitations. First, the cross-sectional design of the study prevents the establishment of causal relationship. Second, although trained interviewers administered depression/anxiety assessment, no physician verified participants’ depression/anxiety status. This could result in respondents falsely reflecting their psychological well-being. Third, the study solely focused on demographic and lifestyle factors, neglecting other viable confounding variables. Unmeasured and residual confounders could influence findings. Finally, FFQ is subject to memory biases and measurement errors, inevitably leading to misclassifications in dietary intake—a common bias in nutritional epidemiology studies. Our study exhibits notable strengths. It encompasses a large, multi-center sample of Iranian adults, enhancing the external validity of our findings. By focusing on healthy subjects, we eliminated the confounding influence of diseases on mental health status. The completion of all questionnaires by trained interviewers enhanced the reliability and precision of responses. Finally, in our assessment of the efficacy of a plant-based diet, we employed a novel eating plan (10) that still needs to undergo evaluation regarding its impact on mental health.

In conclusion, our investigation illuminates a null association between ELD and depression and anxiety in the whole population. However, in stratified analysis by sex, males with higher adherence had lower risk of depression. This research sheds light on ELD potential impact on mental health. There is a scarcity of research on this topic, leading to conflicting results; thus, further studies employing diverse study designs and more robust methodologies are essential to clarify the exact influence of ELD on mental disorders and to explore the underlying mechanisms involved.

## Data Availability

The original contributions presented in the study are included in the article/Supplementary material, further inquiries can be directed to the corresponding author.
