# Supplementary material for: Adherence to the Eat-Lancet diet and its association with depression and anxiety among Iranian adults: a cross-sectional multicentric study
Source: Front Nutr. 2025 Mar 27;12:1524652. doi: 10.3389/fnut.2025.1524652 (PMC11983546; doi:10.3389/fnut.2025.1524652)
Supplement: Supplementary file 1 [file Table_1.docx]

**Supplementary Table 1 Construction of the EAT-Lancet diet score (51)^a^**

| Food component | Subcomponent | Cut-off |
| --- | --- | --- |
| Whole grains |  | ≤ 464 g/d |
| Potatoes and tuber |  | ≤ 100 g/d |
| Vegetables |  | ≤ 200 g/d |
| Fruits |  | ≤ 100 g/d |
| Dairy foods |  | ≤ 500 g/d |
| Protein sources |  |  |
|  | Beef, lamb, pork | ≤ 28 g/d |
|  | Chicken and poultry | ≤ 58 g/d |
|  | Eggs | ≤ 25 g/d |
|  | Fish | ≤ 100 g/d |
|  | Legumes | ≤ 100 g/d |
|  | Nuts | ≤ 25 g/d |
| Added fats |  |  |
|  | Saturated oil | ≤ 11.8 g/d |
|  | Unsaturated oils | ≤ 80 g/d |
| Added sugars | All sweet | ≤ 31 g/d |

^a^ Cut-offs for a 2500 Kcal diet based on Knuppel et al. (Knuppel et al., 2019).

**Supplementary Table 2** Multivariable-adjusted OR and 95% CI for the association of Eat-Lancet diet and severity of depression and anxiety

|  | **Eat-lancet diet (N=1994)** | | | **p trend** |
| --- | --- | --- | --- | --- |
| **Depression** | **Low**  **(n=1608)** | **Medium**  **(n=224)** | **High**  **(n=121)** |  |
| Crude^2^ | 1 | 0.98 (0.74, 1.28) | 0.92 (0.70, 1.22) | 0.577 |
| Model 1 | 1 | 0.90 (0.68, 1.19) | 0.74 (0.56, 0.99) | 0.040 |
| Model 2 | 1 | 0.93 (0.70, 1.24) | 0.76 (0.56, 1.02) | 0.071 |
| **Anxiety** | **Low**  **(n=1545)** | **Medium**  **(n=235)** | **High**  **(n=181)** |  |
| Crude^2^ | 1 | 1.07 (0.83, 1.40) | 1.04 (0.80, 1.35) | 0.762 |
| Model 1 | 1 | 0.92 (0.70, 1.21) | 0.81 (0.62, 1.07) | 0.193 |
| Model 2 | 1 | 0.92 (0.70, 1.22) | 0.80 (0.60, 1.06) | 0.111 |

*Values are OR and 95% CI and resulted from ordinal logistic regression

† Model 1: Adjusted for age, sex, energy intake.

Model 2: Adjusted as for model 1 plus marital status (category), smoking (category), physical activity, socioeconomic status and center effect.
